# Impact of The Affordable Care Act’s Elimination of Cost-Sharing on the Guideline-Concordant Utilization of Cancer Preventive Screenings in the United States Using Medical Expenditure Panel Survey

**DOI:** 10.3390/healthcare7010036

**Published:** 2019-03-01

**Authors:** Naleen Raj Bhandari, Chenghui Li

**Affiliations:** Division of Pharmaceutical Evaluation and Policy, University of Arkansas for Medical Sciences, Little Rock, AR 72205, USA; nrbhandari@uams.edu

**Keywords:** affordable care act, cost-sharing, cancer screening, racial disparity, out-of-pocket payment, mammography, Pap test, colorectal cancer screening, FOBT, colonoscopy

## Abstract

Currently available evidence regarding the association of the Affordable Care Act’s (ACA) elimination of cost-sharing and the utilization of cancer screenings is mixed. We determined whether the ACA’s zero cost-sharing policy affected the guideline-concordant utilization of cancer screenings, comparing adults (≥21 years) from 2009 with 2011–2014 data from the Medical Expenditure Panel Survey. Study participants were categorized as: 21–64 years with any private insurance, ≥65 years with Medicare only, and 21–64 years uninsured, with a separate sample for each type of screening test. Adjusted weighted prevalence and prevalence ratios (PR (95%CI)) were estimated. In 2014 (vs. 2009), privately-insured women reported 2% (0.98 (0.97–0.99)) and 4% (0.96 (0.93–0.99)) reduction in use of Pap tests and mammography, respectively. Privately-insured non-Hispanic Asian women had 16% (0.84 (0.74–0.97)) reduction in mammography in 2014 (vs. 2009). In 2011 (vs. 2009), privately-insured and Medicare-only men reported 9% (1.09 (1.03–1.16)) and 13% (1.13 (1.02–1.25)) increases in colorectal cancer (CRC) screenings, respectively. Privately-insured women reported a 6–7% rise in 2013–2014 (vs. 2009), and Hispanic Medicare beneficiaries also reported 40–44%, a significant rise in 2011–2014 (vs. 2009), in the utilization of CRC screenings. While the guideline-concordant utilization of Pap tests and mammography declined in the post-ACA period, the elimination of cost-sharing appeared to have positively affected CRC screenings of privately-insured males, females, and Hispanic Medicare-only beneficiaries. Greater awareness about the zero cost-sharing policy may help in increasing the uptake of cancer screenings.

## 1. Introduction

Preventive care can reduce overall healthcare costs and improve patient well-being. Although the evidence is mixed on whether use of cancer preventive screenings for cervical cancer (Papanicolaou, or Pap, test), breast cancer (mammography), colorectal cancer (CRC), blood stool tests (FOBT), colonoscopies, or sigmoidoscopies reduces all-cause mortality, screenings for CRC and breast cancer have been shown to reduce disease-specific mortality [1,2,3,4]. Regardless, these screening tests are recommended by the United States Preventive Services Task Force (USPSTF) for eligible populations in the United States.

Patient behavior of utilizing preventive healthcare services could be influenced by the presence or absence of patient cost-sharing, i.e., out-of-pocket payments (OOP) in the form of copays or deductibles [5,6,7,8,9]. The literature, to a great extent, suggests that the amount of OOP negatively affects the use of recommended screenings for breast and cervical cancers [8,9]. To increase the uptake of preventive healthcare services, the Affordable Care Act (ACA), signed into law in the US in March 2010, greatly emphasized disease prevention. According to the ACA’s Preventive Care Provision, beginning September 23, 2010, all non-grandfathered-in private health insurance plans were required to provide coverage for the USPSTF recommended preventive care services without patient cost-sharing [10]. Beginning January 2011, Medicare was also required to cover the USPSTF recommended preventive care services without patient cost-sharing [10]. Additionally, starting in January 2014, the ACA prohibited non-grandfathered-in plans to have annual or lifetime dollar limits for “essential health benefits,” which included preventive care services.

Literature from the pre-ACA period suggests a negative [11,12,13,14,15] or lack of [16,17] association of OOP and utilization of cancer screenings. For instance, Medicare beneficiaries reported lower utilization of mammography when enrolled in health plans with copayments compared to those in plans without copayments and/or those with additional supplemental coverage which protected patients from copayments [11,12,13]. Similarly, individuals aged 18–64 years reported lower utilization of Pap tests and mammography when enrolled in plans with patient cost-sharing compared to others [14,15]. However, Medicare beneficiaries [11] or health maintenance organization enrollees [16] in the pre-ACA period reported no difference in the utilization of Pap tests when enrolled in health plans with/without copayments. Likewise, a study by Han and colleagues in the post-ACA period found no overall increase in utilization of Pap tests, mammography, or screenings for CRC between the years 2009 and 2011–2012 [18]. In individuals aged 50–64 years, Richman et al. reported no effect on the utilization of screenings for CRC in the post-ACA period (i.e., 2012 vs. 2009) [19]. Jenssen et al. also did not find any effects of the elimination of cost-sharing on the utilization of FOBT, prostate-specific antigen tests, and mammography in Medicare beneficiaries in 2008–2010 versus 2012 [20]. Another recent study did not show any differences in the utilization of cancer screenings before and after the implementation of the ACA [21]. However, some studies have also demonstrated a positive effect of zero cost-sharing on the utilization of cancer screenings. For instance, in a sub-group analysis by Han and colleagues, women without chronic conditions reported a small increase in the use of mammography in the post-ACA period [18]. Richman et al. also reported a 12% increase in colonoscopy use among Medicare beneficiaries aged 65–75 years in 2012 (vs. 2009) [19]. More recently, a cancer registry-based study reported an 8% increase in diagnoses of CRC cases in the post-ACA period [22]. Overall, the above-presented studies are limited by relatively early and inconsistent findings.

Disparities in receipt of cancer screenings by race/ethnicity have also been extensively reported [23,24,25,26,27,28,29,30]. According to the currently available literature, Black Americans are equally or more likely to report utilization of screenings for breast and cervical cancers compared to their White counterparts [23,26,27,30], which is also true among those who are uninsured [25]. The evidence about the utilization of screening for CRC is mixed among Black and White Americans [23,26,31]. While in a few studies [23,26,29,30] Hispanics, Asian-Americans, and Pacific Islanders reported lower utilization of screenings for CRC, breast, and cervical cancers compared to other groups, in more recent studies Hispanic women had a higher likelihood of reporting utilization of screenings for breast cancer [24] and cervical cancer [25] compared to non-Hispanic (nH) White women. Additionally, the removal of cost-sharing was positively correlated with the receipt of mammography among a national sample of rural women [32]. Limited evidence also found differences in the uptake of CRC screenings [33]. 

A recent systematic review concluded, the available evidence regarding cost-sharing and utilization of cancer screening in the ACA era is mixed, with a limited number of studies evaluating the short-term effects [34]. However, none of the studies included in the review reported the impact of ACA on cancer screenings by race/ethnicity characteristics [34], despite the well-known disparities in healthcare utilization (including cancer screenings) by race/ethnicity in the US [23,24,25,26,27,28,29,30]. Moreover, most studies in the post-ACA period defined the receipt of cancer screening as those received “within past year,” which is inconsistent with the frequency of screening tests as recommended by the USPSTF, necessitating the need for further studies.

This study examined the association between the ACA’s elimination of cost-sharing and the utilization of USPSTF-recommended preventive screenings for cervical cancer, breast cancer, and CRC, stratified by the type of insurance, race/ethnicity, and sex (for CRC screenings). As opposed to the currently available literature, this study adds to the research by providing the updated prevalence of the guideline-concordant utilization of cancer screenings in the overall eligible population, males, females, as well as within specific racial/ethnic groups, using a nationally representative sample in the US.

## 2. Materials and Methods

### 2.1. Data Source

Household Component (HC) data of the Medical Expenditure Panel Survey (MEPS) was used for this study. Pooled samples from 2009 (pre-ACA period) and 2011–2014 (post-ACA period) of MEPS full-year data were used. The year 2010 was excluded, because the ACA was enacted in that year. MEPS is sponsored by the Agency for Healthcare Research and Quality, and collects information about the use and expenditures of healthcare services, demographic characteristics, and health status from a nationally representative, civilian, non-institutionalized US sample [35]. The calculated average annual response rate was: 53.9% (2009-57.2%, 2011-54.9%, 2012-56.3%, 2013-52.8%, and 2014-48.5%). Further information about MEPS is available from http://meps.ahrq.gov/mepsweb/.

### 2.2. Study Participants

For each year, adults aged ≥21 years were identified and further categorized into three groups based on their age and insurance coverage: (a) participants aged 21–64 with any private insurance, (b) participants aged ≥65 with Medicare only, and (c) participants aged 21–64 with no insurance (uninsured). To maintain compliance with the USPSTF guidelines and the ACA’s Preventive Care Provision, separate age-gender appropriate study groups for each type of cancer preventive screening were defined (Appendix A). Women aged 21–65, excluding those who had hysterectomy or history of cervical cancer, comprised the group for cervical cancer screenings (Pap test), women aged ≥40 without prior history of breast cancer comprised the group for breast cancer screenings (mammography), and participants aged 50–75 without prior history of colon cancer comprised the group for CRC screening (fecal occult blood test, sigmoidoscopy, colonoscopy). Participants with missing responses for preventive cancer screening services were excluded (i.e., 6.4% of those eligible for cervical cancer screening, 7.6% of those eligible for breast cancer screening, and 4.6% of those eligible for CRC screening).

### 2.3. Study Measures—Outcomes

Self-reported receipt of cancer screening was assessed annually based on the following question: “when did (PERSON) have (PERSON)’s the most recent test (pap or mammography or blood stool test (FOBT) or colonoscopy or sigmoidoscopy)?” Among those eligible to receive each screening, binary variables for the receipt of each recommended cancer screening was defined as follows (Appendix A): (a) Pap test—within past 3 years or less, (b) mammography—within past 2 years or less, and (c) any screening for CRC: FOBT—within past year, colonoscopy—within past 10 years or less, or sigmoidoscopy—within past 5 years or less. These definitions were consistent with the most recent USPSTF recommendations during the years studied: 2012 (cervical cancer [36]), 2002 and 2009 (breast cancer [37,38]) and 2008 (colorectal cancer [39]) (Appendix A). In 2009, because the USPSTF breast cancer screening recommendations increased the starting age to 50 years, as opposed to 40 years (in 2002), two mammography use variables were defined using each recommendation. The updated 2012 cervical cancer screening guidelines also recommended Pap tests every five years if combined with human papillomavirus (HPV) testing among women aged 30–65 years. However, HPV testing information is unavailable from MEPS and was not measured.

### 2.4. Study Measures—Independent Variable of Interest

The main independent variable of interest was “survey year”, coded as 2009 (pre-ACA period), 2011, 2012, 2013, and 2014 (post-ACA periods).

### 2.5. Study Measures—Covariates

Participant demographic characteristics included: age, sex, race/ethnicity, education, employment status, family income, region, and number of chronic conditions. For each participant, a count variable for the number of chronic conditions was created based on the responses to the question: “(Have/Has) (PERSON) ever been told by a doctor or other health professional that (PERSON) had the condition? (yes/no)” The chronic conditions included were arthritis, asthma, hypertension, high cholesterol, diabetes, emphysema, stroke, and other heart-related conditions (angina, myocardial infarction, coronary heart disease, unspecified other).

### 2.6. Statistical Analysis

Participant characteristics were described and compared across years using chi-square tests. A generalized linear model with a log link and Poisson distribution was used to assess the association between the receipt of cancer screening and the survey year, and adjusted prevalence and prevalence ratios were estimated [40]. Regression models, stratified by type of insurance coverage, adjusted for age, sex (CRC screening only), race/ethnicity, education, family income, marital status, region, and number of other chronic conditions. Furthermore, to determine this association within the groups of race/ethnicity and sex, stratification by race/ethnicity (Hispanic, nH White, nH Black, and nH Asian) was conducted for all types of screening groups, and by males and females for the CRC screening group. Prevalence ratios with 95% confidence intervals for each post-ACA period (2011–2014) were compared to the pre-ACA period (2009) to evaluate the association between the ACA’s elimination of cost-sharing and receipt of cancer preventive screenings. All analyses were conducted using Stata/IC 14.2 (College Station, TX, USA) and SASv9.3 (SAS Institute Inc, Cary, NC, USA) with a significance level of 0.05. The MEPS complex survey design and nonresponses were accounted by the survey procedures, and nationally representative weighted estimates were computed [41]. Participants with zero or negative weights were excluded from the regression models.

## 3. Results

A total of 84,274 adult participants (≥21 years) were included in the study, of which 16,700 (19.8%) belonged to the pre-ACA period. Approximately 75% of participants in each year were <65 years of age. The majority of the participants were female, nH White, with some college or bachelor’s degree education, employed, married, privately-insured, had high family income, and categorized into the southern census region. More than 33% of participants in each year reported zero chronic conditions, while another ~22% participants in each year had three or more self-reported chronic conditions. Participant distribution across the studied years differed statistically significantly regarding their age, sex, race/ethnicity, education, insurance coverage, and family income (Table 1).

### 3.1. Screening for Cervical Cancer

The prevalence of the guideline-concordant utilization of Pap tests tended to decline over time (pre- to post-ACA period) across privately insured and uninsured groups, while it tended to increase in women with Medicare-only insurance (Table 2). These trends are also similar across women of different race/ethnicity (Table 4). A statistically significant 2% (*p* < 0.05) decline in receipt of Pap tests was observed in privately-insured women in 2014 vs. 2009 (Table 3). However, it was not specific to a particular type of race/ethnicity (Table 5). Hispanic women with Medicare-only insurance had a 92% (*p* < 0.05) rise in receipt of Pap tests in 2011 compared to 2009 (Table 5).

### 3.2. Screening for Breast Cancer

The prevalence of mammography utilization consistent with the 2002 USPSTF recommendations tended to decline over time across privately-insured and uninsured women (Table 2) across all the race/ethnicity types (Table 4). Compared to 2009, a statistically significant 4% (*p* < 0.05) drop in receipt of mammography (2002) was observed among women with private insurance in 2014 (Table 3). This drop was 16% (*p* < 0.05) in nH Asian women with private insurance when race/ethnicity groups were analyzed separately (Table 5). In the Medicare-only group, the prevalence declined in 2009–2013 (66–63%), but also rose up to 68% in 2014 (Table 2) similarly within each type of race/ethnicity groups (Table 4). However, there were no statistically significant differences between the pre- and post-ACA periods (Table 3 and Table 5). Observations were similar when mammography was defined based on 2009 USPSTF recommendations.

### 3.3. Any Screening for Colorectal Cancer

The prevalence of the guideline-concordant utilization of any screenings for CRC tended to increase over time across privately-insured and Medicare-only groups, while being stable with a spike in 2012 among the uninsured group (Table 2). Specifically, the increase was 6–5% for privately-insured participants and 9–8% for Medicare-only beneficiaries in the post-ACA periods (vs. 2009, Table 3). Further stratification by sex and race/ethnicity revealed more variations (Table 4). Compared to 2009, privately-insured males reported statistically significant 9% (2011, *p* < 0.05) and 12% (2012, *p* < 0.05) increases in screenings for CRC in post-ACA periods (Table 5). In privately-insured females, the increase in screenings for CRC in post-ACA periods was 7% (2013, *p* < 0.05) and 6% (2014, *p* < 0.05) compared to the pre-ACA period (Table 5). Such increases were also observed in Hispanic (15% in 2012), nH white (6% in 2012–2013), nH black (10% in 2013), and nH Asian (24% in 2014) privately-insured participants in post-ACA periods compared to 2009 (*p* < 0.05, Table 5). Among the Medicare-only group, a 13% rise in 2011 (vs. 2009) was statistically significant in males. While there was a rise in utilization of CRC screening among female Medicare-only beneficiaries, it was not statistically significant in 2011–2014 (vs. 2009, Table 5). Hispanic Medicare-only beneficiaries reported a statistically significant (*p* < 0.05) rise in utilization of CRC screenings in all years of the post-ACA period compared to 2009 (Table 5): 40% (2011), 28% (2012), 33% (2013), and 44% (2014).

Although unexpected, uninsured females reported a statistically significant (*p* < 0.05) rise in CRC screenings in 2012 (29%) and 2014 (31%) compared to 2009 (Table 5).

## 4. Discussion

In this study, we evaluated whether the ACA’s elimination of a cost-sharing provision for preventive care services influenced the USPSTF-concordant utilization of selected cancer screenings. To provide a comprehensive report, we stratified the analysis by three insurance groups, and also by race/ethnicity for all screenings and by sex for CRC screenings. Our results suggest the following: (a) utilization of Pap tests in the post-ACA period has generally declined in privately-insured women, however, it has increased in Hispanic Medicare-only women; (b) utilization of mammography in the post-ACA period has declined in privately-insured women where the reduction is greatest in nH Asians, though there are no differences in the uptake of mammography in the Medicare-only group; and (c) utilization of CRC screenings has increased in males and females of all race/ethnicities with private insurance in the post-ACA periods, while its utilization has increased in Hispanic Medicare-only beneficiaries, regardless of sex.

The declining trend in the utilization of recommended Pap tests in privately-insured women in this study is in line with recent data from the Centers for Disease Control and Prevention [29]. However, it is in contrast to previous studies from the pre- and post-ACA periods, which demonstrated negative [14,15] or a lack of [11,21] association between cost-sharing and the uptake of Pap tests. A reason for the decline in Pap tests, specifically after 2012, could be due to the updated guidelines. According to the new updates, women could have a Pap test every five years if they had it in combination with the HPV test [36]. However, the unavailability of information about HPV testing in MEPS data limited our ability to confirm this reasoning. On the Medicare-only side, the elimination of cost-sharing appeared to benefit the Hispanic population, as we observed an increase in the use of Pap tests in the early post-ACA period (2011). However, in the other post-ACA periods, the rise in utilization was not statistically significant.

The stable-declining trend in the uptake of mammography is also in accordance with recent reports [29,42,43]. Fedewa and colleague’s analysis of the National Health Interview Survey reported a 3.2% decline in mammography in 2013 (vs. 4% in 2014 this study) compared to pre-ACA periods in privately-insured women [42]. Even the 16% reduction in mammography screenings among privately-insured nH Asians in this study is similar to a previous report [23]. One of the well-known hypotheses for the reduction in rates of mammography is that women no longer visit physicians for hormone therapy prescriptions—which is usually the visit in which they are referred to mammography—because of a greater risk of breast cancer with use of hormone therapy [44,45,46]. Consistent with previous studies [18,20,42,47], we did not find significant differences in rates of mammography among Medicare-only beneficiaries across pre-ACA and post-ACA periods. 

The findings regarding the rise in CRC screenings in this study are consistent with recent studies by Fedewa et al [42]. and Richman et al. [19]. However, they are in contrast to the study by Cooper et al [48], which reported on only colonoscopies among ≥70 year-old Medicare beneficiaries. Other reports from the Department of Health and Human Services also suggest an overall increase in the use of preventive care services by Medicare beneficiaries [49,50]. However, our observation of an increase in CRC screenings across all years of post-ACA periods was limited to Hispanic Medicare-only beneficiaries. The rise in utilization of screenings for CRC in the post-ACA period observed in this study among privately-insured individuals could, in part, also be attributed to the newly gained coverage among those who were previously uninsured. Given the simultaneous implementation of the ACA to mandate/expand insurance and the elimination of cost-sharing associated with preventive services, it is difficult to isolate the effects of each of these changes.

The extent of awareness about the ACA’s zero cost-sharing requirements for preventive care services is poor, which may have resulted in the decreased utilization of preventive care services observed in this study. According to a recent survey, only less than half of the Americans were aware of this policy [51]. Thus, there is a need to create awareness about the benefits of such a policy. Further, the grandfathered-in plans were not required to implement the zero cost-sharing policy. However, close to 50% participants in the Kaiser survey still reported having grandfathered-in plans [51]. Creating more awareness about the zero cost-sharing provision to both policyholders and clinicians would help in improving the screening rates. Finally, personal factors such as beliefs and perceptions about screening and its associated risks could also play a role in explaining the declining trends observed in this study.

While there are strengths to our study, such as the use of nationally representative data and the USPSTF-concordant definitions for receipt of cancer screenings, there are also some limitations. Responses to receipt of cancer screening were based on self-reported data, which are prone to recollection or recall bias [52], as well as underreporting of utilization [53]. However, we expect that any extent of recall and underreporting would have been similar across the study years, potentially not affecting the results in our study. Further, due to lack of information from the MEPS, we were unable to differentiate between grandfathered-in and non-grandfathered-in private plans. Lastly, the co-occurring policy changes (such as the change in guidelines for mammography and the addition of HPV testing for cervical cancer), and other concurrent events that influenced the survival of the ACA, could have affected our findings and subsequent interpretation.

## 5. Conclusions

In conclusion, our analysis suggests a stable-to-declining trend in the guideline-concordant utilization of screenings for cervical cancer and breast cancer. The ACA’s elimination of a cost-sharing policy appeared to have positively affected CRC screenings of privately-insured males, females, and Medicare-only insured Hispanics. Greater awareness about the zero cost-sharing policy may help in increasing the uptake of cancer screenings.

## Figures and Tables

**Table 1:** Demographic Characteristics of the Study Participants.

Characteristics	Medical Expenditure Panel Survey Year, Weighted Column % (SE)					*p*-Value
	2009 ^†^	2011 ^†^	2012 ^†^	2013 ^†^	2014 ^†^	
**Age, years**						<0.0001
21–25	6.6 (0.3)	7.0 (0.3)	6.9 (0.3)	6.4 (0.3)	6.4 (0.3)	
26–29	5.7 (0.3)	5.2 (0.2)	5.2 (0.2)	5.3 (0.3)	5.2 (0.2)	
30–39	12.5 (0.3)	12.3 (0.3)	12.4 (0.3)	12.5 (0.3)	12.4 (0.4)	
40–49	14.9 (0.3)	13.5 (0.3)	13.4 (0.4)	13.0 (0.3)	12.5 (0.3)	
50–64	39.2 (0.6)	39.7 (0.6)	39.4 (0.6)	39.6 (0.6)	39.7 (0.6)	
65–75	15.0 (0.4)	16.4 (0.5)	16.9 (0.5)	17.5 (0.5)	17.8 (0.5)	
>75	6.1 (0.3)	5.9 (0.3)	5.7 (0.3)	5.7 (0.3)	5.9 (0.3)	
**Sex**						0.026
Male	26.1 (04)	26.9 (0.4)	26.9 (0.4)	27.4 (0.4)	27.5 (0.4)	
Female	73.9 (0.4)	73.1 (0.4)	73.1 (0.4)	72.6 (0.4)	72.5 (0.4)	
**Race/Ethnicity**						<0.0001
Hispanic	11.7 (0.7)	12.8 (0.8)	13.0 (0.8)	13.0 (0.7)	13.7 (0.8)	
Non-Hispanic White	70.6 (0.9)	68.8 (1.0)	68.3 (1.0)	67.9 (1.0)	66.5 (1.1)	
Non-Hispanic Black	11.3 (0.6)	11.4 (0.7)	11.6 (0.6)	11.5 (0.7)	11.7 (0.6)	
Non-Hispanic Asian	4.5 (0.4)	5.1 (0.5)	5.1 (0.5)	5.5 (0.4)	5.6 (0.5)	
Non-Hispanic Other	1.8 (0.2)	1.9 (0.2)	1.9 (0.2)	2.1 (0.2)	2.4 (0.3)	
**Education**						<0.0001
High School or Less	44.5 (0.7)	40.1 (0.7)	30.9 (0.6)	37.6 (0.6)	36.9 (0.7)	
Some College or Bachelor′s Degree	43.0 (0.6)	46.5 (0.6)	55.4 (0.5)	50.1 (0.5)	50.5 (0.6)	
Master′s Degree or Higher	12.5 (0.5)	13.4 (0.5)	13.6 (0.5)	12.2 (0.5)	12.6 (0.5)	
**Employment Status**						0.472
Employed	62.4 (0.6)	62.2 (0.7)	61.7 (0.6)	62.2 (0.6)	63.0 (0.6)	
Unemployed	37.5 (0.6)	37.8 (0.7)	38.3 (0.6)	37.8 (0.6)	37.0 (0.6)	
**Marital Status**						0.492
Married	59.0 (0.7)	58.1 (0.7)	57.7 (0.7)	58.1 (0.7)	58.1 (0.8)	
Unmarried	41.0 (0.7)	41.9 (0.7)	42.3 (0.7)	41.9 (0.7)	41.9 (0.8)	
**Insurance Coverage**						<0.0001
Aged 21–64 years, Any Private	72.4 (0.7)	72.7 (0.8)	71.1 (0.8)	70.6 (0.8)	74.0 (0.8)	
Aged 65+ years, Medicare	12.3 (0.4)	12.6 (0.5)	13.3 (0.5)	13.6 (0.5)	13.9 (0.5)	
Aged 21–64 years, Uninsured	15.3 (0.5)	14.7 (0.5)	15.6 (0.6)	15.8 (0.6)	12.1 (0.5)	
Family Income *						0.02
Low	29.2 (0.7)	30.4 (0.7)	30.2 (0.7)	30.2 (0.7)	29.8 (0.7)	
Medium	29.3 (0.6)	29.3 (0.6)	29.2 (0.6)	28.6 (0.6)	27.2 (0.6)	
High	41.5 (0.8)	40.3 (0.8)	40.6 (0.9)	41.2 (0.8)	43.0 (0.9)	
**Region ^‡^**						0.999
Northeast	18.7 (0.8)	18.6 (0.7)	18.6 (0.7)	18.6 (0.7)	18.1 (0.8)	
Midwest	22.0 (0.8)	21.6 (0.7)	21.6 (0.7)	21.7 (0.7)	21.7 (0.8)	
South	36.6 (0.9)	37.2 (0.9)	37.3 (0.9)	36.8 (0.9)	37.4 (1.0)	
West	22.7 (0.8)	22.6 (0.7)	22.5 (0.7)	22.9 (0.6)	22.8 (0.7)	
**Number of Chronic Conditions ^^^**						0.128
Zero	34.3 (0.6)	31.2 (0.5)	34.5 (0.5)	44.0 (0.6)	33.3 (0.6)	
One	22.8 (0.4)	22.6 (0.5)	22.5 (0.4)	21.8 (0.4)	22.4 (0.5)	
Two	17.6 (0.4)	17.5 (0.4)	17.9 (0.4)	18.1 (0.4)	17.7 (0.4)	
Three or more	25.3 (0.5)	24.7 (0.5)	25.1 (0.5)	26.1 (0.5)	26.6 (0.6)	

Estimates are adjusted for Medical Expenditure Panel Survey (MEPS) survey design and nonresponse. ^†^ Unweighted N: 16,700 (2009), 16,337 (2011), 18,018 (2012), 17,036 (2013), 16,183 (2014). * Family Income: Low (<200% Federal Poverty Level (FPL)), Medium (200–400% FPL) and High (>400% FPL). ^‡^ Northeastern states (Connecticut, Maine, Massachusetts, New Hampshire, New Jersey, New York, Pennsylvania, Rhode Island, and Vermont), Midwestern States (Indiana, Illinois, Iowa, Kansas, Michigan, Minnesota, Missouri, Nebraska, North Dakota, Ohio, South Dakota, and Wisconsin), Southern States (Alabama, Arkansas, Delaware, District of Columbia, Florida, Georgia, Kentucky, Louisiana, Maryland, Mississippi, North Carolina, Oklahoma, South Carolina, Tennessee, Texas, Virginia, and West Virginia), Western States (Alaska, Arizona, California, Colorado, Hawaii, Idaho, Montana, Nevada, New Mexico, Oregon, Utah, Washington, and Wyoming). ^^^ Chronic conditions included were: arthritis, asthma, hypertension, high cholesterol, diabetes, emphysema, stroke, and other heart-related conditions (angina, myocardial infarction, coronary heart disease, unspecified other). Abbreviations: SE-standard error.

**Table 2:** Adjusted Prevalence of Self-Reported Receipt of Cancer Screenings in Pre- and Post-Affordable Care Act (ACA) Periods, Stratified by Insurance.

Screening	Medical Expenditure Panel Survey Year, Adjusted Prevalence (95% CI)				
	2009	2011	2012	2013	2014
**Aged 21–64 years, Any Private Insurance**					
Pap Test	0.91 (0.90, 0.92)	0.92 (0.91, 0.93)	0.90 (0.89, 0.91)	0.89 (0.88, 0.91)	0.89 (0.88, 0.90)
Mammography (2002)	0.79 (0.77, 0.81)	0.79 (0.77, 0.81)	0.80 (0.78, 0.81)	0.77 (0.75, 0.79)	0.76 (0.74, 0.78)
Mammography (2009)	0.82 (0.80, 0.84)	0.83 (0.81, 0.85)	0.83 (0.81, 0.85)	0.82 (0.80, 0.84)	0.81 (0.78, 0.83)
Any CRC Screening	0.59 (0.57, 0.61)	0.62 (0.60, 0.64)	0.63 (0.61, 0.66)	0.63 (0.61, 0.65)	0.62 (0.60, 0.64)
**Aged ≥65 years, Medicare-only**					
Pap Test	0.73 (0.57, 0.89)	0.77 (0.62, 0.92)	0.77 (0.58, 0.96)	0.76 (0.58, 0.94)	0.76 (0.59, 0.93)
Mammography (2002)	0.66 (0.63, 0.69)	0.63 (0.59, 0.66)	0.64 (0.60, 0.68)	0.63 (0.59, 0.67)	0.68 (0.65, 0.71)
Mammography (2009)	0.75 (0.71, 0.79)	0.73 (0.68, 0.78)	0.76 (0.71, 0.82)	0.71 (0.67, 0.76)	0.76 (0.72, 0.81)
Any CRC Screening	0.67 (0.63, 0.71)	0.73 (0.70, 0.76)	0.72 (0.68, 0.76)	0.71 (0.67, 0.74)	0.72 (0.69, 0.76)
**Aged 21–64 years, Uninsured**					
Pap Test	0.75 (0.72, 0.77)	0.75 (0.73, 0.77)	0.74 (0.72, 0.77)	0.72 (0.70, 0.75)	0.72 (0.68, 0.75)
Mammography (2002)	0.45 (0.42, 0.49)	0.46 (0.42, 0.50)	0.48 (0.44, 0.53)	0.42 (0.38, 0.45)	0.42 (0.37, 0.47)
Mammography (2009)	0.47 (0.42, 0.51)	0.52 (0.46, 0.58)	0.54 (0.48, 0.59)	0.45 (0.40, 0.50)	0.49 (0.42, 0.55)
Any CRC Screening	0.28 (0.25, 0.31)	0.28 (0.25, 0.32)	0.33 (0.29, 0.37)	0.28 (0.24, 0.31)	0.28 (0.24, 0.32)

Prevalence and 95% confidence intervals in each row are obtained from separate regression models. Each model was weighted and adjusted for MEPS survey design, nonresponse, age, race/ethnicity, education, family income, marital status, region, and number of other chronic conditions. Mammography was defined based on USPSTF 2002 & 2009 recommendations separately. Abbreviations: CRC- colorectal cancer, USPSTF- United States Preventive Services Task Force.

**Table 3:** Adjusted Prevalence Ratios Representing the Association of Medical Expenditure Panel Survey Year with Self-Reported Receipt of Cancer Screenings, Stratified by Insurance.

Screening	Medical Expenditure Panel Survey Year, Adjusted Prevalence Ratio (95% CI)				
	2009	2011	2012	2013	2014
**Aged 21–64 years, Any Private Insurance**					
Pap Test	Ref	1.01 (0.99, 1.02)	0.99 (0.97, 1.01)	0.98 (0.96, 1.00)	0.98 (0.97, 0.99) *
Mammography (2002)	Ref	1.00 (0.97, 1.03)	1.00 (0.97, 1.04)	0.98 (0.95, 1.01)	0.96 (0.93, 0.99) *
Mammography (2009)	Ref	1.01 (0.97, 1.05)	1.01 (0.98, 1.05)	1.00 (0.96, 1.03)	0.98 (0.94, 1.02)
Any CRC Screening	Ref	1.06 (1.01, 1.10) *	1.07 (1.03, 1.12) *	1.07 (1.02, 1.11) *	1.05 (1.00, 1.09) *
**Aged ≥65 years, Medicare-only**					
Pap Test	Ref	1.06 (0.80, 1.41)	1.06 (0.76, 1.49)	1.05 (0.76, 1.44)	1.04 (0.77, 1.42)
Mammography (2002)	Ref	0.95 (0.88, 1.03)	0.97 (0.90, 1.04)	0.95 (0.87, 1.03)	1.03 (0.96, 1.10)
Mammography (2009)	Ref	0.97 (0.90, 1.05)	1.02 (0.94, 1.10)	0.95 (0.88, 1.04)	1.02 (0.94, 1.10)
Any CRC Screening	Ref	1.09 (1.02, 1.16) *	1.07 (0.99, 1.15)	1.06 (0.99, 1.13)	1.08 (1.01, 1.15) *
**Aged 21–64 years, Uninsured**					
Pap Test	Ref	1.00 (0.96, 1.05)	1.00 (0.94, 1.05)	0.97 (0.93, 1.02)	0.96 (0.91, 1.01)
Mammography (2002)	Ref	1.01 (0.90, 1.13)	1.06 (0.95, 1.18)	0.92 (0.81, 1.04)	0.93 (0.81, 1.06)
Mammography (2009)	Ref	1.11 (0.96, 1.30)	1.15 (1.01, 1.32) *	0.96 (0.82, 1.12)	1.04 (0.88, 1.24)
Any CRC Screening	Ref	1.02 (0.88, 1.20)	1.19 (1.02, 1.38) *	1.01 (0.86, 1.19)	1.03 (0.86, 1.22)

* *p* < 0.05. Prevalence Ratios and 95% confidence intervals in each row are obtained from separate regression models. Each model was weighted and adjusted for MEPS survey design, nonresponse, age, race/ethnicity, education, family income, marital status, region, and number of other chronic conditions. Mammography was defined based on USPSTF 2002 & 2009 recommendations separately.

**Table 4:** Adjusted Prevalence of Self-Reported Receipt of Cancer Screenings in Pre- and Post-ACA Periods, Stratified by Insurance, Race/Ethnicity, and Sex.

Screening	Medical Expenditure Panel Survey Year, Adjusted Prevalence (95% CI)				
	2009	2011	2012	2013	2014
**Aged 21–64 years, Any Private Insurance**					
**Pap Test**					
Hispanic	0.90 (0.87, 0.92)	0.92 (0.90, 0.94)	0.92 (0.89, 0.94)	0.89 (0.87, 0.91)	0.91 (0.90, 0.93)
Non-Hispanic White	0.91 (0.90, 0.93)	0.92 (0.91, 0.93)	0.90 (0.89, 0.91)	0.89 (0.88, 0.91)	0.89 (0.88, 0.91)
Non-Hispanic Black	0.94 (0.92, 0.95)	0.95 (0.93, 0.97)	0.94 (0.92, 0.95)	0.93 (0.91, 0.95)	0.93 (0.91, 0.95)
Non-Hispanic Asian	0.85 (0.81, 0.89)	0.87 (0.82, 0.91)	0.85 (0.81, 0.89)	0.81 (0.77, 0.85)	0.80 (0.75, 0.84)
**Mammography (2002)**					
Hispanic	0.78 (0.74, 0.83)	0.77 (0.71, 0.83)	0.77 (0.73, 0.82)	0.77 (0.73, 0.82)	0.77 (0.73, 0.81)
Non-Hispanic White	0.79 (0.77, 0.81)	0.80 (0.77, 0.82)	0.80 (0.78, 0.82)	0.77 (0.74, 0.79)	0.76 (0.73, 0.78)
Non-Hispanic Black	0.83 (0.79, 0.86)	0.84 (0.80, 0.88)	0.82 (0.78, 0.85)	0.81 (0.78, 0.84)	0.82 (0.79, 0.85)
Non-Hispanic Asian	0.79 (0.72, 0.85)	0.72 (0.65, 0.79)	0.78 (0.72, 0.84)	0.71 (0.65, 0.77)	0.66 (0.59, 0.73)
**Any CRC Screening**					
Male	0.58 (0.56, 0.61)	0.64 (0.61, 0.66)	0.65 (0.62, 0.68)	0.62 (0.59, 0.64)	0.60 (0.57, 0.63)
Female	0.60 (0.57, 0.62)	0.61 (0.58, 0.64)	0.62 (0.59, 0.65)	0.64 (0.61, 0.67)	0.63 (0.60, 0.66)
Hispanic	0.49 (0.44, 0.54)	0.54 (0.49, 0.59)	0.57 (0.52, 0.61)	0.55 (0.50, 0.59)	0.54 (0.50, 0.59)
Non-Hispanic White	0.61 (0.58, 0.63)	0.64 (0.61, 0.66)	0.64 (0.62, 0.67)	0.64 (0.62, 0.66)	0.63 (0.60, 0.65)
Non-Hispanic Black	0.61 (0.57, 0.66)	0.66 (0.63, 0.70)	0.66 (0.61, 0.71)	0.68 (0.64, 0.71)	0.64 (0.60, 0.69)
Non-Hispanic Asian	0.44 (0.37, 0.51)	0.45 (0.38, 0.53)	0.52 (0.46, 0.58)	0.50 (0.43, 0.57)	0.54 (0.48, 0.60)
**Aged ≥65 years, Medicare-only**					
**Pap Test**					
Hispanic	0.47 (0.19, 0.76)	0.91 (0.60, 1.22)	0.56 (0.10, 1.23)	0.84 (0.48, 1.20)	0.68 (0.42, 0.94)
Non-Hispanic White	0.71 (0.47, 0.95)	0.69 (0.47, 0.90)	0.81 (0.56, 1.06)	0.70 (0.39, 1.01)	0.91 (0.64, 1.18)
Non-Hispanic Black	0.88 (0.61, 1.15)	0.89 (0.73, 1.06)	1.11 (0.60, 1.61)	0.87 (0.70, 1.03)	0.76 (0.55, 0.96)
Non-Hispanic Asian	Model Did not Converge				
**Mammography (2002)**					
Hispanic	0.65 (0.57, 0.73)	0.67 (0.59, 0.75)	0.68 (0.58, 0.77)	0.70 (0.64, 0.76)	0.74 (0.68, 0.79)
Non-Hispanic White	0.67 (0.63, 0.71)	0.62 (0.57, 0.67)	0.65 (0.60, 0.69)	0.63 (0.58, 0.68)	0.68 (0.63, 0.72)
Non-Hispanic Black	0.71 (0.64, 0.78)	0.71 (0.65, 0.78)	0.66 (0.57, 0.74)	0.63 (0.56, 0.69)	0.69 (0.63, 0.75)
Non-Hispanic Asian	0.51 (0.34, 0.67)	0.51 (0.38, 0.65)	0.48 (0.33, 0.62)	0.56 (0.44, 0.68)	0.59 (0.48, 0.70)
**Any CRC Screening**					
Male	0.67 (0.61, 0.73)	0.76 (0.71, 0.80)	0.75 (0.70, 0.80)	0.73 (0.68, 0.78)	0.73 (0.68, 0.78)
Female	0.67 (0.62, 0.71)	0.71 (0.67, 0.79)	0.69 (0.64, 0.74)	0.70 (0.65, 0.74)	0.72 (0.68, 0.76)
Hispanic	0.51 (0.42, 0.61)	0.72 (0.65, 0.79)	0.65 (0.55, 0.75)	0.69 (0.62, 0.75)	0.74 (0.68, 0.80)
Non-Hispanic White	0.71 (0.66, 0.76)	0.75 (0.71, 0.79)	0.75 (0.70, 0.80)	0.73 (0.68, 0.77)	0.73 (0.69, 0.77)
Non-Hispanic Black	0.65 (0.57, 0.72)	0.70 (0.63, 0.76)	0.64 (0.56, 0.72)	0.74 (0.68, 0.80)	0.72 (0.66, 0.79)
Non-Hispanic Asian	0.53 (0.39, 0.66)	0.52 (0.37, 0.68)	0.50 (0.35, 0.66)	0.53 (0.41, 0.64)	0.63 (0.48, 0.77)
**Aged 21–64 years, Uninsured**					
**Pap Test**					
Hispanic	0.82 (0.79, 0.86)	0.78 (0.75, 0.82)	0.79 (0.75, 0.83)	0.80 (0.77, 0.83)	0.77 (0.73, 0.81)
Non-Hispanic White	0.69 (0.64, 0.73)	0.70 (0.66, 0.75)	0.70 (0.65, 0.74)	0.66 (0.61, 0.71)	0.67 (0.61, 0.74)
Non-Hispanic Black	0.80 (0.76, 0.85)	0.86 (0.81, 0.91)	0.83 (0.78, 0.87)	0.82 (0.77, 0.88)	0.75 (0.68, 0.83)
Non-Hispanic Asian	0.60 (0.48, 0.72)	0.59 (0.47, 0.71)	0.64 (0.55, 0.74)	0.57 (0.47, 0.67)	0.58 (0.43, 0.73)
**Mammography (2002)**					
Hispanic	0.58 (0.53, 0.64)	0.55 (0.50, 0.60)	0.54 (0.46, 0.62)	0.54 (0.48, 0.60)	0.54 (0.47, 0.61)
Non-Hispanic White	0.38 (0.32, 0.43)	0.37 (0.31, 0.43)	0.43 (0.36, 0.49)	0.33 (0.27, 0.39)	0.31 (0.23, 0.40)
Non-Hispanic Black	0.58 (0.51, 0.65)	0.60 (0.52, 0.69)	0.57 (0.49, 0.65)	0.49 (0.43, 0.55)	0.55 (0.46, 0.64)
Non-Hispanic Asian	0.35 (0.20, 0.49)	0.50 (0.35, 0.64)	0.45 (0.32, 0.59)	0.46 (0.29, 0.62)	0.42 (0.20, 0.64)
**Any CRC Screening**					
Male	0.26 (0.22, 0.30)	0.27 (0.22, 0.32)	0.28 (0.23, 0.34)	0.23 (0.18, 0.28)	0.20 (0.15, 0.25)
Female	0.29 (0.24, 0.33)	0.30 (0.25, 0.35)	0.37 (0.31, 0.43)	0.33 (0.28, 0.38)	0.37 (0.31, 0.44)
Hispanic	0.21 (0.17, 0.26)	0.22 (0.16, 0.27)	0.22 (0.16, 0.28)	0.24 (0.19, 0.28)	0.22 (0.16, 0.29)
Non-Hispanic White	0.31 (0.26, 0.36)	0.30 (0.25, 0.35)	0.36 (0.30, 0.42)	0.30 (0.24, 0.35)	0.29 (0.24, 0.35)
Non-Hispanic Black	0.33 (0.27, 0.39)	0.33 (0.25, 0.41)	0.32 (0.26, 0.38)	0.30 (0.23, 0.37)	0.39 (0.30, 0.47)
Non-Hispanic Asian	Model Did not Converge				

Prevalence and 95% confidence intervals in each row are obtained from separate regression models. Each model for race/ethnicity (all screenings) was weighted and adjusted for MEPS survey design, nonresponse, age, gender (for CRC only), education, family income, marital status, region, and number of other chronic conditions. Each model for gender (CRC only) was weighted and adjusted for MEPS survey design, nonresponse, age, race/ethnicity, education, family income, marital status, region, and number of other chronic conditions. Mammography was defined based on USPSTF 2002 recommendations.

**Table 5:** Adjusted Prevalence Ratios Representing the Association of Medical Expenditure Panel Survey Year with Self-Reported Receipt of Cancer Screenings, Stratified by Insurance, Race/Ethnicity, and Sex.

Screening	Medical Expenditure Panel Survey Year, Adjusted Prevalence Ratio (95% CI)				
	2009	2011	2012	2013	2014
**Aged 21–64 years, Any Private Insurance**					
**Pap Test**					
Hispanic	Ref	1.03 (1.00, 1.06)	1.02 (0.98, 1.06)	1.00 (0.96, 1.03)	1.02 (0.99, 1.05)
Non-Hispanic White	Ref	1.00 (0.98, 1.03)	0.98 (0.96, 1.00)	0.98 (0.96, 1.00)	0.98 (0.96, 1.00)
Non-Hispanic Black	Ref	1.01 (0.98, 1.04)	1.00 (0.97, 1.02)	0.99 (0.97, 1.02)	0.99 (0.97, 1.02)
Non-Hispanic Asian	Ref	1.02 (0.95, 1.09)	1.00 (0.93, 1.07)	0.95 (0.89, 1.02)	0.94 (0.88, 1.00)
**Mammography (2002)**					
Hispanic	Ref	0.98 (0.90, 1.07)	0.99 (0.90, 1.08)	0.99 (0.91, 1.08)	0.98 (0.90, 1.07)
Non-Hispanic White	Ref	1.01 (0.97, 1.05)	1.01 (0.97, 1.05)	0.97 (0.94, 1.01)	0.96 (0.91, 1.00)
Non-Hispanic Black	Ref	1.02 (0.96, 1.08)	0.99 (0.93, 1.05)	0.98 (0.93, 1.04)	1.00 (0.94, 1.05)
Non-Hispanic Asian	Ref	0.91 (0.82, 1.02)	0.99 (0.90, 1.10)	0.90 (0.79, 1.02)	0.84 (0.74, 0.97) *
**Any CRC Screening**					
Male	Ref	1.09 (1.03, 1.16) *	1.12 (1.05, 1.18) *	1.06 (1.00, 1.13)	1.03 (0.97, 1.10)
Female	Ref	1.02 (0.96, 1.09)	1.03 (0.97, 1.10)	1.07 (1.01, 1.13) *	1.06 (1.00, 1.12) *
Hispanic	Ref	1.10 (0.97, 1.24)	1.15 (1.00, 1.32) *	1.11 (0.97, 1.26)	1.10 (0.97, 1.25)
Non-Hispanic White	Ref	1.05 (0.99, 1.10)	1.06 (1.01, 1.12) *	1.06 (1.00, 1.11) *	1.03 (0.98, 1.09) *
Non-Hispanic Black	Ref	1.08 (0.99, 1.17)	1.07 (0.97, 1.18)	1.10 (1.01, 1.19) *	1.04 (0.96, 1.13)
Non-Hispanic Asian	Ref	1.04 (0.82, 1.01)	1.19 (0.98, 1.16)	1.13 (0.91, 1.41)	1.24 (1.03, 1.49) *
**Aged ≥65 years, Medicare-only**					
**Pap Test**					
Hispanic	Ref	1.92 (1.06, 3.47) *	1.19 (0.32, 4.39)	1.77 (0.79, 3.96)	1.43 (0.81, 2.53)
Non-Hispanic White	Ref	0.97 (0.62, 1.52)	1.14 (0.72, 1.80)	0.99 (0.59, 1.66)	1.28 (0.81, 2.04)
Non-Hispanic Black	Ref	1.02 (0.69, 1.51)	1.26 (0.77, 2.06)	0.98 (0.68, 1.42)	0.86 (0.53, 1.40)
Non-Hispanic Asian	Model Did not Converge				
**Mammography (2002)**					
Hispanic	Ref	1.03 (0.89, 1.20)	1.04 (0.87, 1.24)	1.07 (0.91, 1.26)	1.13 (0.96, 1.32)
Non-Hispanic White	Ref	0.93 (0.84, 1.03)	0.97 (0.89, 1.06)	0.95 (0.85, 1.06)	1.02 (0.93, 1.11)
Non-Hispanic Black	Ref	1.00 (0.87, 1.16)	0.92 (0.78, 1.09)	0.88 (0.77, 1.01)	0.97 (0.84, 1.12)
Non-Hispanic Asian	Ref	1.02 (0.68, 1.53)	0.94 (0.62, 1.42)	1.11 (0.76, 1.63)	1.16 (0.79, 1.71)
**Any CRC Screening**					
Male	Ref	1.13 (1.02, 1.25) *	1.12 (1.00, 1.25)	1.08 (0.97, 1.20)	1.09 (0.98, 1.21)
Female	Ref	1.07 (0.98, 1.16)	1.03 (0.93, 1.14)	1.04 (0.94, 1.15)	1.07 (0.98, 1.17)
Hispanic	Ref	1.40 (1.14, 1.72) *	1.28 (1.00, 1.61) *	1.33 (1.10, 1.62) *	1.44 (1.18, 1.76) *
Non-Hispanic White	Ref	1.06 (0.98, 1.15)	1.05 (0.96, 1.15)	1.02 (0.94, 1.11)	1.03 (0.95, 1.12)
Non-Hispanic Black	Ref	1.08 (0.93, 1.24)	0.99 (0.85, 1.15)	1.14 (1.00, 1.30)	1.12 (0.98, 1.27)
Non-Hispanic Asian	Ref	0.99 (0.68, 1.46)	0.95 (0.64, 1.41)	1.00 (0.73, 1.36)	1.19 (0.84, 1.69)
**Aged 21–64 years, Uninsured**					
**Pap Test**					
Hispanic	Ref	0.95 (0.90, 1.00)	0.96 (0.91, 1.01)	0.97 (0.93, 1.01)	0.93 (0.89, 0.98) *
Non-Hispanic White	Ref	1.03 (0.93, 1.13)	1.01 (0.91, 1.12)	0.96 (0.87, 1.07)	0.98 (0.87, 1.11)
Non-Hispanic Black	Ref	1.08 (1.00, 1.16) *	1.03 (0.96, 1.11)	1.03 (0.94, 1.12)	0.94 (0.83, 1.06)
Non-Hispanic Asian	Ref	0.97 (0.72, 1.32)	1.06 (0.84, 1.35)	0.95 (0.73, 1.23)	0.96 (0.70, 1.31)
**Mammography (2002)**					
Hispanic	Ref	0.95 (0.86, 1.06)	0.93 (0.81, 1.07)	0.92 (0.82, 1.04)	0.93 (0.80, 1.08)
Non-Hispanic White	Ref	0.98 (0.80, 1.22)	1.14 (0.93, 1.41)	0.87 (0.67, 1.13)	0.84 (0.62, 1.12)
Non-Hispanic Black	Ref	1.04 (0.86, 1.26)	0.99 (0.84, 1.16)	0.85 (0.72, 1.00) *	0.95 (0.77, 1.16)
Non-Hispanic Asian	Ref	1.43 (0.85, 2.42)	1.31 (0.79, 2.19)	1.31 (0.74, 2.33)	1.21 (0.61, 2.39)
**Any CRC Screening**					
Male	Ref	1.04 (0.81, 1.33)	1.09 (0.86, 1.37)	0.89 (0.69, 1.16)	0.77 (0.57, 1.03)
Female	Ref	1.04 (0.84, 1.29)	1.29 (1.04, 1.61) *	1.14 (0.93, 1.40)	1.31 (1.04, 1.65) *
Hispanic	Ref	1.02 (0.74, 1.39)	1.02 (0.71, 1.44)	1.10 (0.84, 1.43)	1.03 (0.72, 1.47)
Non-Hispanic White	Ref	0.98 (0.79, 1.22)	1.18 (0.95, 1.47)	0.97 (0.76, 1.23)	0.96 (0.75, 1.23)
Non-Hispanic Black	Ref	0.99 (0.72, 1.37)	0.97 (0.74, 1.27)	0.92 (0.67, 1.25)	1.17 (0.89, 1.54)
Non-Hispanic Asian	Model Did not Converge				

* *p* < 0.05. Prevalence Ratios and 95% confidence intervals in each row are obtained from separate regression models. Each model for race/ethnicity (all screenings) was weighted and adjusted for MEPS survey design, nonresponse, age, gender (for CRC only), education, family income, marital status, region and number of other chronic conditions. Each model for gender (CRC only) was weighted and adjusted for MEPS survey design, nonresponse, age, race/ethnicity, education, family income, marital status, region, and number of other chronic conditions. Mammography was defined based on USPSTF 2002 recommendations.

## Appendix A

**Table A1:** Recommended frequency of preventive screenings for cancer by USPSTF and study definitions for compliance and receipt of cancer screening used in this study.

Type of Screening	USPSTF Recommendations			Study Definition for Compliance & Receipt of Type of Screening
	Grade (Year of Recommendation)	Age Range	Screening Frequency	
Cervical Cancer Screening (Pap Test) ^1^	A (2012)	21–65 years	Every 3 years	Within past 3 years or less
Breast Cancer Screening (Mammography) ^2^	B (2002)	≥40 years	Every 1–2 years	Within past 2 years or less
Breast Cancer Screening (Mammography) ^3^	B (2009)	50–74 years	Every 2 years	Within past 2 years or less
Colorectal Cancer Screening ^4^	A (2008)	50–75 years		
FOBT (Blood Stool Test using Home Kit)			Every year	within past year
Colonoscopy			Every 10 years	within past 10 years or less
Sigmoidoscopy			Every 5 years	within past 5 years or less

^1^https://www.uspreventiveservicestaskforce.org/Page/Document/UpdateSummaryFinal/cervical-cancer-screening. ^2^https://www.uspreventiveservicestaskforce.org/Page/Document/UpdateSummaryFinal/breast-cancer-screening-2002. ^3^https://www.uspreventiveservicestaskforce.org/Page/Document/UpdateSummaryFinal/breast-cancer-screening. ^4^https://www.uspreventiveservicestaskforce.org/Page/Document/UpdateSummaryFinal/colorectal-cancer-screening.
